# Genomic Characterization of Aerobically Culturable Gut-Associated Bacteria and Yeasts Isolated from Pooled Larval Midguts of the Lesser Mealworm *Alphitobius diaperinus* (Coleoptera: Tenebrionidae)

**DOI:** 10.3390/insects17080800

**Published:** 2026-08-02

**Authors:** Gisele Ivonne Antonuccio, Pablo Julián López, Marcelo Facundo Berretta, Diego Herman Sauka

**Affiliations:** 1 Instituto Nacional de Tecnología Agropecuaria (INTA), Instituto de Microbiología y Zoología Agrícola (IMYZA), Buenos Aires 1686, Argentina; lopez.julian@inta.gob.ar (P.J.L.); berretta.marcelo@inta.gob.ar (M.F.B.); sauka.diego@inta.gob.ar (D.H.S.); 2 Comisión de Investigaciones Científicas (CIC), Gobierno de la Provincia de Buenos Aires, La Plata 1900, Argentina; 3 Consejo Nacional de Investigaciones Científicas y Técnicas (CONICET), Ciudad Autónoma de Buenos Aires 1425, Argentina

**Keywords:** microbial ecology, comparative genomics, polyphasic approach, culturable larval gut microbiota, *Alphitobius diaperinus*, functional annotation

## Abstract

Microbial communities associated with insect larvae play key roles in host nutrition, protection against pathogens, immune modulation, and represent a source of biotechnologically relevant traits. In *Alphitobius diaperinus*, a major poultry pest, the gut microbiota has been mainly studied at the community level, providing limited insight into the potential roles of individual microorganisms. Here, we characterized the aerobically culturable fraction of the larval gut microbiota derived from a single pooled homogenate of fifteen larvae under stable laboratory rearing conditions—an approach that uniquely enables strain-level genomic resolution, functional experimentation, and biotechnological exploitation. Ten bacterial strains and two yeasts were initially isolated; eight bacterial isolates were identical, revealing the most frequently recovered cultivable bacterial morphotype. We provide high-quality draft genomes for five members of the *A. diaperinus* cultivable gut microbiota—*Enterobacter hormaechei* subsp. *xiangfangensis* strain INTA AN1-1, *Staphylococcus hominis* subsp. *novobiosepticus* strain INTA AC1-4, *Staphylococcus succinus* subsp. *succinus* strain INTA AC1-8, *Hyphopichia burtonii* strain INTA AB1-1, and *Debaryomyces fabryi* strain INTA AB1-4—establishing a genome-resolved reference framework validated through a polyphasic approach. Genome-scale annotation enabled us to predict the metabolic potential and hypothesize the functional capabilities among cultivable members, providing a foundation for future studies on this economically relevant pest.

## Simple Summary

Advances in genome sequencing allow the identification of bacteria and yeasts with high taxonomic resolution, providing insights into microbial ecology, predicted metabolic potential, and functional roles beyond traditional morphology- or biochemistry-based methods. In this study, we investigated the culturable fraction of the gut microbiota derived from a single pooled sample of fifteen larvae of the lesser mealworm, *Alphitobius diaperinus*, a key pest in poultry production, under limited aerobic conditions. A total of twelve isolates were initially obtained (ten bacteria and two yeasts), but eight bacterial isolates were identical, so a single representative was selected. Using Illumina whole-genome sequencing, we identified three bacterial strains and two yeasts. Although this culturable fraction represents a minor proportion of the total gut community it offers a critical advantage over culture-independent approaches: these isolates can be maintained under laboratory conditions, enabling direct functional experiments, strain-level genomic resolution and biotechnological exploitation. No novel species were detected, yet our results reveal microorganisms with distinct functional profiles isolated in the larval gut. This work establishes a genome-resolved reference framework for the cultivable bacteria and yeasts isolates obtained from healthy *A. diaperinus* larvae in the present study and provides genomic resources for future ecological and functional investigations.

## 1. Introduction

Understanding the ecological relevance of the gut microbiota in insect larvae requires considering the functional contributions of gut-associated microorganisms. In insects, bacterial communities are known to support host nutrition, protection against parasites and pathogens, modulation of immune responses, and intra- and interspecific communication [1]. In contrast, intestinal yeasts remain comparatively understudied despite playing similarly important roles [2]. Characterizing the culturable fraction of insect gut microbiota provides an opportunity to integrate microbial ecology with genome-resolved analyses, enabling the recovery of physical isolates for downstream functional and comparative studies.

This limitation is particularly evident in the lesser mealworm, *Alphitobius diaperinus* (Coleoptera: Tenebrionidae). Native to Africa, this holometabolous insect has spread worldwide, including to Argentina, where it has become a key pest in poultry production systems, directly impacting production efficiency, causing structural damage to poultry house facilities, and generating allergic reactions and irritation in farm workers [3]. Despite extensive research on *A. diaperinus* pest management and biology, knowledge of its gut-associated microbiota remains limited, particularly for cultivable microorganisms during the larval stage.

A careful survey of the literature demonstrates that Cucini et al., 2020 [4] investigated the bacterial and fungal diversity of the gut microbiota of *A. diaperinus* larvae fed polystyrene. They reported bacterial genera such as *Cronobacter*, *Kocuria*, and *Pseudomonas*, as well as fungal taxa including *Aspergillus*, *Hyphodermella*, and *Trichoderma*, providing evidence that both bacteria and fungi are present in the intestinal tract of *A. diaperinus* under specific dietary conditions. In a complementary study focusing on adult *A. diaperinus*, Crippen et al., 2022 [5], using 16S rDNA analysis, reported that the most abundant bacterial phyla in individuals collected from broiler houses were Proteobacteria (39.8%), followed by Firmicutes (30.8%), Actinobacteria (21.1%), Tenericutes (5.1%), and Bacteroidetes (1.6%). Additional insight into the gut-associated microbiota of *A. diaperinus* was provided by Crippen et al., 2022 [6], who examined how management practices influence the microbial community of lesser mealworm larvae in broiler production systems. Using 16S rRNA gene sequencing, this study characterized the bacterial community profile of larvae collected from a broiler grow-out house and following relocation of the litter to pastureland. The larval gut microbiota was dominated by members of the phyla Tenericutes, Actinobacteria, Bacteroidetes, Firmicutes, and Proteobacteria. More recently, Ndotono et al., 2024 [7] investigated the gut microbiota of *A. diaperinus* larvae in the context of polystyrene consumption in Kenya. In their study, the predominant bacterial genera observed in larvae fed polystyrene-based diets were *Kluyvera*, *Lactococcus*, *Klebsiella*, *Enterobacter*, and *Enterococcus*, whereas *Stenotrophomonas* dominated in larvae maintained on a control diet.

Collectively, these investigations demonstrate that to date, most available studies rely on 16S rRNA (ribosomal ribonucleic acid) gene sequencing, metabarcoding, or metagenomic approaches, which provide community-level or taxonomically broad insights but lack species-resolved, genome-based characterization of individual gut-associated microorganisms. As a result, the microbial ecology of the larval gut, including the functional potential of cultivable taxa, remains largely unexplored [4,5,6,7,8].

To address this knowledge gap, this study presents draft genome sequences of five cultivable gut-associated microorganisms isolated from *A. diaperinus* larvae reared under stable laboratory conditions (Microbial Inputs Laboratory, IMYZA–INTA Castelar, Buenos Aires, Argentina). The objective of this study was to isolate and characterize the aerobic culturable fraction of the gut microbiota of *A. diaperinus* larvae through a polyphasic approach integrating phenotypic characterization—including colonial and cellular morphology, Gram staining, scanning electron microscopy, and biochemical profiling—with whole-genome sequencing (WGS), enabling high-resolution taxonomic identification supported by integrated phenotypic and genomic data.

On the basis of these considerations, we propose the following hypotheses: whole-genome sequencing permits reliable taxonomic identification beyond conventional phenotypic and/or 16S rRNA and ITS gene analyses alone; phenotypic and genomic profiles of isolates show congruence, validating the integrated polyphasic approach; and genome-based functional characterization provides comprehensive metabolic predictions, revealing functional potential not accessible through partial gene amplification.

## 2. Materials and Methods

### 2.1. Isolation and Enumeration

The Instituto de Microbiología y Zoología Agrícola (IMYZA) at the Instituto Nacional de Tecnología Agropecuaria (INTA) in Hurlingham, Buenos Aires, Argentina, has established a unique laboratory rearing system for *A. diaperinus* for research purposes, which, to the best of our knowledge, is the only one of its kind in the country. All developmental stages were maintained on a commercial chick starter feed (Ganave^®^), routinely used in poultry production systems. The colony was established in 2012 from specimens originally collected from poultry production facilities in the region and maintained at the Universidad Nacional de Luján, Buenos Aires, Argentina. Prior to incorporation into our laboratory, all specimens underwent quarantine and were confirmed to be free of pests. The colony has been maintained continuously since 2012 without new external inputs. Rearing conditions were: temperature 28 °C, relative humidity 60–70%, and photoperiod 14 h light: 10 h darkness. Under these conditions, *A. diaperinus* is multivoltine, producing at least four generations annually. Due to cannibalistic behavior, larvae, pupae, and adults were maintained separately in a dedicated incubator. Oviposition substrates were replaced every 48–72 h, and containers were regularly disinfected and sieved to remove dust generated by feeding.

To isolate the cultivable gut microbiota from healthy *A. diaperinus* larvae, fifteen seventh-instar larvae were subjected to a 24 h starvation period and then underwent surface disinfection. Larval midguts were then dissected and pooled into a single composite sample. This disinfection procedure involved a series of steps, including immersion in 70% ethanol, exposure to a 15% bleach solution, and three rinses in physiological solution, following the protocol outlined by Lu et al., 2013 [9]. The third rinse served as a control before proceeding with the dissection of the midguts. The fifteen midguts were homogenized together in 250 μL of physiological solution using a sterile mortar. To obtain serial dilutions, 100 μL of the homogenate was transferred to an Eppendorf tube containing 900 μL of physiological solution (10-fold dilution), and this procedure was repeated sequentially until reaching a final dilution of 10^−6^. We inoculated 100 μL of four dilutions (ranging from 10^−3^ to 10^−6^), each in quintuplicate, on nutrient agar (NA) and brain heart infusion agar (BHI). Additionally, 100 μL of the undiluted midgut suspension was inoculated in triplicate on *Bacillus cereus* agar (BC). Broad-spectrum culture media were primarily used to maximize the recovery of physiologically robust cultivable microorganisms for downstream ecological interpretation, while the selective BC medium was included to facilitate the detection of spore-forming *Bacillus*-like colonies.

The plates were then incubated for 72 h at 28 °C. Selections of colonies displaying distinct morphologies were isolated from the various culture media until pure cultures were obtained, facilitating subsequent identification processes.

Quantification of colony-forming units (CFUs). Colony counts were performed on plates displaying 30–300 colonies, following the acceptable colony-count range for quantification. For each dilution and medium type, the average number of colonies from replicate plates was calculated. The number of CFUs per gut was estimated using the standard formula: (mean colony count × dilution factor)/inoculum volume (mL).

### 2.2. Phenotypic and Microscopic Characterization

Gram staining was performed on all culturable strains. Scanning electron microscopy (SEM) was subsequently used to examine five selected cultivable gut-associated isolates from the total obtained. Representative cells from each isolate were examined at the Museo Argentino de Ciencias Naturales Bernardino Rivadavia using a Zeiss GeminiSEM 360 microscope (Oberkochen, Germany). Micrographs were obtained under high-vacuum conditions to document cellular morphology and surface features of bacterial and yeast isolates.

### 2.3. Preliminary Identification of Bacterial and Yeast Isolates Based on Partial 16S rRNA, ITS Sequences and Biochemical Characterization

The isolated bacteria were identified at the genus level by amplifying and subsequently sequencing the 16S rRNA gene using primers 27F (5′-AGAGTTTGATCMTGGCTCAG-3′) and 1492R (5′-TACGGYTACCTTGTTACGACTT-3′) as described by Weisburg et al., 1991 [10]. For yeast identification, we amplified and sequenced a fragment comprising the internal transcribed spacer (ITS) 1, the 5.8S rRNA gene, and ITS 2, along with a segment of the large subunit rRNA gene, utilizing primers ITS1 (5′-TCCGTAGGTGAACCTGCGG-3′) and ITS4 (5′-TCCTCCGCTTATTGATATGC-3′) following the protocol outlined by White et al., 1990 [11].

For bacterial amplification, PCR reactions were performed in a final volume of 50 μL containing: 34 μL sterile water, 5 μL 10× reaction buffer, 1 μL dNTP mixture (10 mM), 1 μL primer 27F (10 μM), 1 μL primer 1492R (10 μM), 2.5 μL MgCl_2_ (25–50 mM), 0.5 μL Taq DNA polymerase (Invitrogen, Waltham, MA, USA, 5 U/μL), and 5 μL genomic DNA template. The thermal cycling program consisted of an initial denaturation step at 94 °C for 2 min, followed by 35 cycles of denaturation at 94 °C for 40 s, primer annealing at 55 °C for 40 s, and extension at 72 °C for 1 min 50 s, with a final extension at 72 °C for 10 min. Expected amplicon length was approximately 1600–1700 bp. For yeast amplification, PCR reactions were performed in a final volume of 50 μL containing: 33.8 μL sterile water, 5 μL 10× reaction buffer, 1 μL dNTP mixture (10 mM), 1 μL primer ITS1 (10 μM), 1 μL primer ITS4 (10 μM), 3 μL MgCl_2_ (25–50 mM), 0.2 μL Taq DNA polymerase (Invitrogen, 5 U/μL), and 5 μL genomic DNA template. The thermal cycling program consisted of an initial denaturation step at 94 °C for 1 min, followed by 30 cycles of denaturation at 94 °C for 15 s, primer annealing at 58 °C for 15 s, and extension at 72 °C for 15 s. Expected amplicon lengths ranged from approximately 400 to 600 bp.

PCR product purification was performed using the Wizard SV Gel and PCR Clean-up System (Promega, Madison, WI, USA) following the manufacturer’s instructions. PCR product quality was assessed by 1.5% agarose gel electrophoresis to confirm the presence of expected amplicon sizes.

The PCR products underwent sequencing using the Big Dye Terminator v3.1 Cycle Sequencing Kit and were subsequently analyzed on the ABI PRISM 3100 Genetic Analyzer Sequencer (Applied Biosystems, Hitachi, Tokyo, Japan). Sequence alignment was carried out using the NCBI web BLAST interface v2.13 (Basic Local Alignment Search Tool Nucleotide, National Center for Biotechnology Information) with default search parameters and compared against sequences from reference strains available in the GenBank database (http://www.ncbi.nlm.nih.gov/genbank/, accessed on 3 March 2023). Phylogenetic analyses were performed using MEGA11 (Molecular Evolutionary Genetics Analysis).

Following this initial identification, the bacterial isolates were further identified to the species level by employing bioMérieux API systems (Marcy-l’Étoile, France), in addition to other conventional biochemical tests. API 20E and API 20A for INTA AN1-1, AN1-5, AN1-10, AN1-15, AC1-3, AC1-6, AC1-9 and AC1-14 isolates; and API STAPH for INTA AC1-4 and AC1-8 isolates. The selection of API 20E and API STAPH was based on preliminary characterization, including morphology, phenotypic traits, and PCR results. Although API 20A is designed for anaerobic bacteria, it was applied to the same eight aerobic isolates characterized with API 20E to expand the biochemical dataset and generate a more comprehensive phenotypic profile. All incubations were performed overnight at 29 °C, except for INTA AC1-4, which was incubated at 37 °C according to its growth requirements. Results were read and interpreted at 24 h following the manufacturer’s guidelines. Biochemical identification was used exclusively as preliminary phenotypic characterization; definitive taxonomic assignments were based on whole-genome sequencing analyses, as described in Section 2.3.

### 2.4. Whole-Genome Sequencing

Pure cultures of five representative microorganisms recovered from the culturable fraction of the larval gut of *A. diaperinus* were used in this study, corresponding to those described in Section 2.2, including three bacterial isolates (INTA AN1-1, INTA AC1-4, and INTA AC1-8) and two yeasts (INTA AB1-1 and INTA AB1-4). These five isolates were selected from the twelve obtained in total based on their morphological distinctiveness and preliminary biochemical profiles, in order to capture the breadth of the culturable microbiota while prioritizing genomic characterization of the most divergent representatives. Among the eight Gram-negative isolates identified as *Enterobacter* sp. based on 16S rRNA gene sequencing, a single representative (INTA AN1-1) was randomly selected for whole-genome sequencing, while the remaining isolates with distinct taxonomic identifications were all included. Fresh subcultures were obtained by streaking isolates onto appropriate solid media and incubating overnight at 29 °C, except for isolate INTA AC1-4, which was incubated at 37 °C.

Single, well-isolated colonies from overnight plates were inoculated into sterile nutrient broth and incubated with agitation (250 rpm) at 37 °C using an orbital shaker (Innova 40, New Brunswick Scientific, Edison, NJ, USA). After incubation, liquid cultures were examined by light microscopy to confirm the absence of contamination prior to genomic DNA (Deoxyribonucleic Acid) extraction.

Genomic DNA was extracted from overnight liquid cultures using the Wizard^®^ Genomic DNA Purification Kit (Promega), following the manufacturer’s instructions. DNA quality and concentration were assessed using a NanoDrop™ Thermo Scientific spectrophotometer (Waltham, MA, USA). High-quality DNA was defined as samples with A260/A280 ratios between 1.7 and 1.9 and A260/A230 ratios between 2.0 and 2.2. Only DNA preparations meeting these criteria were used for downstream applications.

Whole-genome sequencing was performed using Illumina technology (San Diego, CA, USA) at the Administración Nacional de Laboratorios e Institutos de Salud Dr. Carlos G. Malbrán (ANLIS–Malbrán). All samples were sequenced on an Illumina NovaSeq 6000 platform using standard short-read workflows, targeting 100× coverage with 150 bp paired-end reads, with bacterial and yeast isolates processed in separate runs. Genomic libraries were prepared using the Illumina DNA Prep kit with dual indexing.

### 2.5. Bioinformatic Workflow for Bacterial Genomes

Raw paired-end FASTQ reads generated at ANLIS–Malbrán were processed using the KBase platform (https://www.kbase.us/). For each bacterial isolate, reads were analyzed within a dedicated narrative. Read quality was assessed with FastQC v0.12.1 [12], followed by adapter removal and quality trimming using Trimmomatic v0.36 [13]. Filtered paired reads were assembled de novo using SPAdes [14], implemented in KBase (v3.15.3) and in a local Linux environment (v4.2.0).

To optimize assembly quality, multiple combinations of trimming parameters and assembly strategies were evaluated. Assemblies were compared based on genome completeness and contamination estimates, prioritizing high completeness and low contamination over contiguity metrics such as the number of contigs. The best-performing assembly for each isolate was selected accordingly, and the optimal combination varied among isolates.

#### 2.5.1. Genome Assembly Quality Assessment and Annotation of Bacterial Isolates

Draft genome assemblies were evaluated to assess assembly quality and suitability for downstream genome-based analyses. Assembly statistics, including the total number of contigs, largest contig size, total assembly length, GC (guanine-cytosine) content, N50 and N90 (the contig length at which 50% and 90% of the total assembly is covered, respectively), and L50 and L90 (the minimum number of contigs needed to reach 50% and 90% of the total assembly length, respectively), were calculated using QUAST v4.4 [15]. Genome completeness and estimated contamination were assessed using CheckM v1.0.18 [16], based on the presence and redundancy of lineage-specific single-copy marker genes. Genome annotation was performed using Prokka v1.15.6 [17] executed in a Linux environment (WSL = Windows Subsystem for Linux), and annotation outputs were used to derive genome annotation statistics, including the number of protein-coding genes, annotated coding sequences (CDS), hypothetical proteins, and RNA genes.

#### 2.5.2. Phylogenomic Analysis and Species Delineation (TYGS)

Phylogenomic analysis of the bacterial isolates was performed using the Type Strain Genome Server (TYGS). For each isolate, TYGS was used to identify the closest related type strains based on genome-wide comparisons. The TYGS pipeline [18] was run with default parameters to support species delineation through genome-based metrics. The results were used to guide the selection of the most closely related type strains for downstream comparative genomic and phylogenomic analyses.

#### 2.5.3. Genome Relatedness Metrics (ANI, dDDH, GC%) and Genome-Based Phylogenetic Reconstruction

Genome relatedness between the bacterial isolates and their closest reference type strains was assessed using multiple genome-wide similarity metrics. Digital DNA–DNA hybridization (dDDH) estimates (d0, d4, and d6) and G+C content differences were obtained using the TYGS pipeline [18]. Average Nucleotide Identity values based on BLAST (ANIb) and MUMmer (ANIm), as well as tetranucleotide correlation coefficients (tetra z-scores), were calculated using JSpecies v3.0 [19]. These metrics were used to support genome-based species delineation.

For phylogenomic inference, genome-based trees were reconstructed using GToTree v1.8.16 [20]. The analysis included the closest related type strains identified by TYGS. Accession numbers for these type strains were retrieved from the List of Prokaryotic names with Standing in Nomenclature (LPSN) [21], and the corresponding genome assemblies were downloaded from GenBank. The phylogenetic tree was built using the Neighbor Joining method with 1000 bootstrap replicates and the Kimura 2-parameter model of nucleotide substitution, with pairwise deletion of gaps/missing data. The resulting phylogenomic trees were visualized and edited using MEGA [22].

#### 2.5.4. Functional Genome Annotation and Feature Inference of Bacterial Isolates

Functional genomic features were inferred from comparative genome annotation of the three bacterial isolates. Predicted protein-coding sequences were functionally annotated using eggNOG-mapper v2.1.8 [23] implemented in the Galaxy platform. The analysis followed a two-step workflow consisting of a search phase to identify seed orthologs, followed by an annotation phase to assign functional categories. Both phases were based on the eggNOG database v5.0.2, and ortholog searches were performed using DIAMOND v2.0.15 [24]. Carbohydrate-active enzymes (CAZymes) were annotated using run_dbcan v5.2.2, the standalone tool of dbCAN (database for Carbohydrate-Active enzyme ANnotation; [25]). Biosynthetic gene clusters were identified using antiSMASH v8.0.4 (antibiotics and Secondary Metabolite Analysis SHell) with default parameters for bacterial genomes [26]. Antimicrobial resistance–associated genes were identified using AMRFinderPlus v4.2.5 with the corresponding AMRFinder database v2025-12-03.1 and were classified as core or acquired according to AMRFinderPlus annotations [27]. To specifically assess potential polymyxin resistance determinants, the presence of acquired colistin resistance genes (*mcr* variants) was screened using the same AMRFinderPlus results. In addition, chromosomal polymyxin-resistance loci (*pmrAB*, *phoPQ*, and *mgrB*) were manually inspected in the genome of isolate INTA AN1-1 by BLAST-based comparison against the reference genome of *E. hormaechei* subsp. *xiangfangensis*. Amino acid sequences of the corresponding regulators were extracted and compared to the reference proteins to identify identical loci or potential substitutions. Virulence-associated genes were identified using ABRicate v1.2.0 with VFDB (Virulence Factor DataBase, last updated 13 January 2026; [28]), applying thresholds of ≥90% sequence identity and ≥90% coverage.

### 2.6. Yeast Bioinformatic Workflow and Genome Annotation

Yeast genomes were processed using a workflow similar to that applied to bacterial isolates. Raw paired-end reads were assembled de novo using SPAdes [14], implemented in KBase (v3.15.3). Contigs shorter than 500 bp were removed. When applicable, alternative trimming and assembly parameter combinations were evaluated, including runs performed in a local Linux environment using SPAdes (v4.2.0). The optimal configuration for each isolate was selected based on CheckM and QUAST results, prioritizing high completeness and low contamination over contiguity metrics. Filtered contigs were mapped with Bowtie2 v2.3.2 [29] against reference genomes of previously identified species within each genus, and the closest related type strain was selected for downstream analyses.

Assembly quality metrics were evaluated with QUAST [15], and repetitive regions were masked using RepeatMasker Galaxy Version 4.1.5+galaxy0 [30] prior to annotation. Genome annotation and functional analyses were conducted using a combination of complementary tools, including BUSCO 6.0.0 (Benchmarking Universal Single-Copy Orthologs), MAKER, InterProScan, OrthoVenn3, and the UNITE ITS pipeline.

ITS sequences of yeast isolates INTA AB1-1 and INTA AB1-4 were extracted from genome assemblies using custom scripts with zero mismatch tolerance. The extracted region spanned from the universal primer ITS1 (5′-TCCGTAGGTGAACCTGCGG-3′) to ITS4 (5′-TCCTCCGCTTATTGATATGC-3′) [31], and the resulting sequences were compared against the UNITE database using https://unite.ut.ee/ [32]. The ITS sequence of strain INTA AB1-1 (443 bp) showed 100% identity with *H. burtonii*, with no mismatches. Similarly, the ITS sequence of strain INTA AB1-4 (639 bp) matched *D. hansenii* with 100% identity, full-length alignment coverage, and no mismatches, thereby excluding the presence of novel species or subspecies among the analyzed isolates.

In addition to the ITS region, the ITS1–NL4 region, extending from the ITS1 primer (5′-TCCGTAGGTGAACCTGCGG-3′; [31]) to the NL4 primer (5′-GGTCCGTGTTTCAAGACGG; [33]), was extracted from genome assemblies using custom scripts with zero mismatch tolerance.

Genome annotation of yeast genomes was performed using MAKER v2.31.11 [34] implemented in the Galaxy platform to estimate the total number of genes. For strain INTA AB1-1, MAKER was run using a masked de novo genome assembly of the isolate together with a *H. burtonii* protein set and the *D. hansenii* model organism. For strain INTA AB1-4, MAKER was run using a masked de novo genome assembly of the isolate together with a *D. fabryi* protein set and the *D. hansenii* model organism. This approach allowed the estimation of the expected total gene number for each yeast isolate.

The total number of Gene Ontology (GO) terms for each yeast isolate was calculated using InterProScan database v5.59-91.0 [35] on the usegalaxy.eu platform. For strains INTA AB1-1 and INTA AB1-4, analyses were performed using as input the MAKER-predicted protein FASTA derived from the de novo genome assembly of each sequenced yeast isolate, together with the protein set of the most closely related type strain (*H. burtonii* or *D. fabryi*, respectively) and the *D. hansenii* prediction model.

InterProScan TSV outputs for the yeast isolates AB1-1 and AB1-4 were parsed using custom Python 3.12.12 scripts. Proteins annotated with the Gene Ontology term GO:0022857 (transmembrane transporter activity) were selected and subjected to a strict keyword-based filtering step to remove false positives, retaining only bona fide transporter-related annotations. The resulting proteins were classified into major functional categories (e.g., MFS = Major Facilitator Superfamily, ABC = ATP-Binding Cassette, sugar, amino acid/peptide, and ion transporters) based on their annotation descriptions. Non-redundant transporter counts were calculated per strain, and summary tables were generated for downstream analysis.

Gut-related and microbial interaction-associated proteins were curated from InterProScan-derived annotations. Proteins were counted once, irrespective of the number of stress-related functional categories assigned. Proteins passing the interaction filter were subsequently classified into functional categories related to adhesion, cell wall/surface anchoring, or biofilm-associated properties based on keyword-driven annotation patterns in InterPro and signature descriptions.

Orthologous clustering was performed in OrthoVenn3 [36] using pairwise comparisons between the predicted proteomes of INTA AB1-1 and the *H. burtonii* type strain, and between INTA AB1-4 and the *D. fabryi* type strain, applying the OrthoMCL algorithm (E-value cutoff 1 × 10^−2^; inflation value 1.5).

CAZyme annotation was performed using dbCAN [25]. Genes were considered CAZymes when supported by at least one detection method (DIAMOND, dbCAN HMM, or dbCAN-sub), consistent with the approach applied to bacterial isolates.

Biosynthetic gene clusters were identified using antiSMASH v8.0.2 (fungiSMASH) with default parameters [26].

#### 2.6.1. Phylogenomic Analysis of Yeast Isolates

Phylogenomic inference was based on single-copy BUSCO [37] orthologs and required the implementation of a dedicated workflow in a Linux environment (WSL). BUSCO analyses were conducted for all ingroup genomes, including all available species of the genera *Hyphopichia* and *Debaryomyces* as well as the newly assembled genomes, using the lineage dataset Debaryomycetaceae_odb12. BUSCO output files were parsed using the scripts evaluate.py and buscolist.py to identify high-confidence shared single-copy orthologs based on predefined average and median thresholds, followed by gene filtering and sequence preprocessing using custom shell scripts. Amino acid sequences were concatenated per genome, aligned using MAFFT (Multiple Alignment using Fast Fourier Transform, [38]) on the Galaxy Europe platform (usegalaxy.eu), and phylogenetic trees were inferred with IQ-TREE multicore v2.4.0 [39]. The resulting Newick tree files were extracted using custom Python scripts and visualized using Biopython 1.86 [40] and Matplotlib 3.10.8 [41].

In addition, a second phylogenetic analysis was performed using the complete ribosomal DNA region spanning from ITS1 to NL4, which was extracted from genome assemblies using custom scripts allowing zero mismatches. The phylogenetic tree was built using the Neighbor Joining method with 1000 bootstrap replicates and the Kimura 2-parameter model of nucleotide substitution, with pairwise deletion of gaps/missing data. This analysis enabled direct comparison with previously obtained phylogenies based on partial ITS–LSU (Large SubUnit rRNA) sequences.

#### 2.6.2. Nucleotide Identity and Divergence Estimation

Pairwise nucleotide identity between the yeast isolates INTA AB1-1 and INTA AB1-4 and their closest related type strains was calculated from genome-wide data using BLASTn [42] on concatenated single-copy BUSCO coding sequences. For each isolate, the corresponding concatenated nucleotide sequence (combinedFastas.fna), derived from complete genome assemblies and filtered to retain single-copy BUSCO genes only, was used as a query and compared against a database comprising concatenated BUSCO nucleotide sequences from all available species within the genera *Hyphopichia* and *Debaryomyces*. BLASTn [42] searches were performed using the megablast algorithm, and results were summarized across all high-scoring segment pairs (HSPs).

Genome-wide nucleotide identity values were calculated as length-weighted averages by summing the product of percent identity and alignment length for each HSP and dividing by the total aligned length. The number of divergent nucleotides was estimated as the product of the total aligned length and the complement of the length-weighted nucleotide identity. All calculations and data processing were performed using custom Python scripts.

In parallel, average nucleotide identity (ANI) was calculated using FastANI [43] to obtain an alignment-free estimate of genome-wide similarity between each isolate and its closest related type strains.

In addition to genome-wide comparisons, pairwise nucleotide identity based on the ribosomal DNA region spanning from ITS1 to NL4 was calculated independently from multiple sequence alignments. ITS1–NL4 sequences were extracted directly from genome assemblies using custom scripts allowing zero mismatches and aligned using Clustal Omega (https://www.ebi.ac.uk/jdispatcher/msa/clustalo, accessed on 30 October 2025) [44]. Percent nucleotide identity was calculated using a pairwise deletion approach, considering only alignment positions where both sequences contained a nucleotide and excluding columns with gaps in either sequence. The number of divergent nucleotides was defined as the number of mismatched positions among these gap-free comparable sites; insertion and deletion events (indels) were not included in divergence estimates.

#### 2.6.3. MAOG-Based Amino Acid Identity and Divergent Amino Acid Estimation

Amino acid identity between the yeast isolates INTA AB1-1 and INTA AB1-4 and their closest related type strains was estimated using a MAOG (Multiple Alignment of Orthologous Genes) -based approach derived from a MAFFT [38] multiple sequence alignment of concatenated conserved single-copy BUSCO protein sequences (combinedFastas.faa), resulting in an alignment of identical length across all taxa. Pairwise amino acid identity was calculated directly from the MAFFT [38] alignment using the identity distance metric implemented in Biopython [40], and pairwise distances (D) were transformed into identity values using the function 1 − D^2^, yielding MAOG-based amino acid identity estimates that reflect divergence within the conserved core proteome. Identity values were expressed as percentages.

The number of divergent amino acid positions was estimated by multiplying the alignment length by the complement of the corresponding MAOG-based amino acid identity value. Analyses focused on pairwise comparisons between each INTA isolate and the most closely related species within the corresponding genus (*Hyphopichia* for INTA AB1-1 and *Debaryomyces* for INTA AB1-4). All calculations, data processing, and visualizations were performed using custom Python scripts.

## 3. Results

### 3.1. Morphological and Phenotypic Characterization of Culturable Fraction from the Larval Gut of A. diaperinus Using CFU Counts, Gram Staining, and SEM

Initial characterization of the cultivable gut microbiota from *A. diaperinus* larvae included colony-forming unit (CFU) counts, Gram staining, and the random selection of representative isolates for further analyses. Higher bacterial counts were observed in BHI (2.51 × 10^5^ CFU/gut) than in NA (2.25 × 10^5^ CFU/gut), likely reflecting the greater nutrient richness of the former. These values should be interpreted as estimates of the cultivable fraction recovered under the conditions employed, rather than direct measures of total microbial abundance; consequently, numerical differences in this particular variable should not be considered as biologically meaningful in the absence of independent biological replication and statistical analysis. Gram staining revealed eight Gram-negative bacilli, identified as recurrently isolated microorganisms of the larval gut cultivable microbiota, and two Gram-positive coccoid isolates recovered from BHI. An unexpected finding was the isolation of two yeasts from macerated intestines inoculated directly, without dilution, onto BC medium, which is typically used for the isolation of Gram-positive bacteria belonging to the *Bacillus cereus* group. Both yeast isolates stained as Gram-positive, with one exhibiting pseudohyphal structures and the other lacking them.

**Figure 1:**
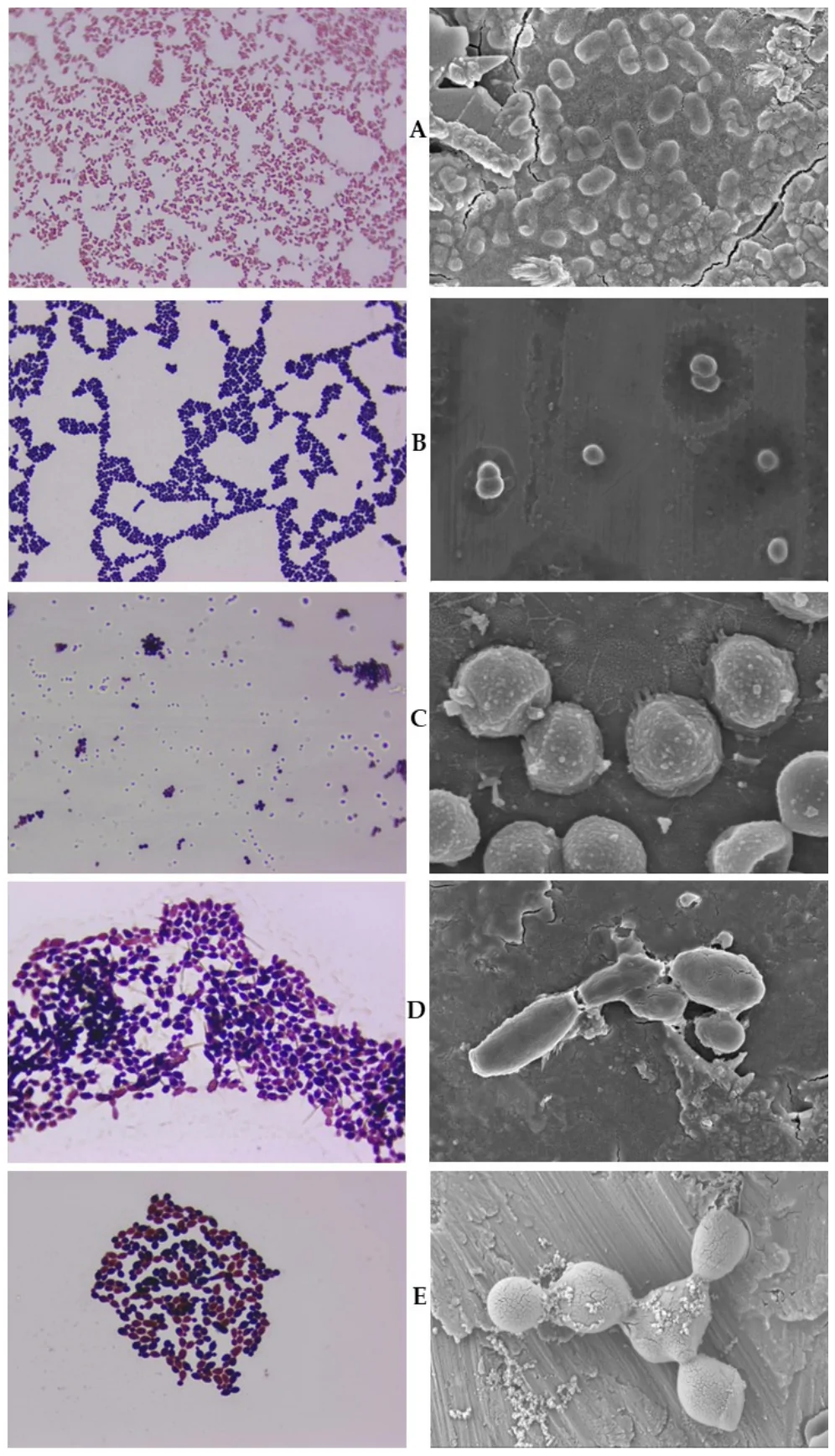
Characterization of culturable gut-associated microorganisms isolated from *A. diaperinus* larvae. For each isolate, the left panel shows Gram staining observed under a 1000× magnification immersion lens, and the right panel shows scanning electron microscopy (SEM) images (7.50; 5.11; 26.96; 8.00 and 6.50 K×, respectively). Isolates were recovered on the following media: Nutrient Agar (**A**), Brain Heart Infusion Agar (**B**,**C**), and *Bacillus cereus* Agar (**D**,**E**). (**A**) *Enterobacter* sp. strain INTA AN1-1. (**B**) *Staphylococcus* sp. strain INTA AC1-4. (**C**) *Staphylococcus* sp. strain INTA AC1-8. (**D**) *Hyphopichia* sp. strain INTA AB1-1. (**E**) *Debaryomyces* sp. strain INTA AB1-4. Two representative micrographs are shown for each isolate at different magnifications. Scale bars and imaging parameters are indicated in each panel.

Scanning electron microscopy (SEM) confirmed the morphological features previously observed by light microscopy in the selected representative isolates. The most abundant bacterial isolate displayed a bacillary morphology with short rod-shaped cells (Figure 1A). Two coccoid morphotypes were observed, both forming irregular clusters of spherical cells, with differences in apparent cell density and aggregation between isolates (Figure 1B,C), likely influenced by sample preparation and cell concentration. The yeast isolates showed distinct morphologies, with one forming pseudohyphae (Figure 1D) and the other consisting exclusively of oval yeast cells without pseudohyphal development (Figure 1E).

### 3.2. Preliminary Identification of Bacterial and Yeast Isolates Based on Partial 16S rRNA, ITS Sequences, and Biochemical Characterization

Phylogenetic analysis based on partial 16S rRNA and ITS sequences allowed genus-level identification of all isolates and comparison with closely related species. The eight Gram-negative bacilli were assigned to *Enterobacter* sp., closely related to *E. hormaechei* subsp. *hormaechei*. The two Gram-positive cocci were identified as *Staphylococcus* spp., one clustering with *S. hominis* (including subsp. *hominis* and subsp. *novobiosepticus*) and the other with S. *succinus* (subsp. *casei* and subsp. *succinus*). ITS sequencing classified the two yeast isolates as *Debaryomyces* sp. and *Hyphopichia* sp., the latter closely related to *H. burtonii*. All sequences have been deposited in GenBank, as detailed in the Data Availability Statement.

In addition, preliminary phenotypic characterization of the bacterial isolates was conducted using API systems and complementary biochemical tests. The isolates INTA AN1-1, INTA AN1-5, INTA AN1-10, INTA AN1-15, INTA AC1-3, INTA AC1-6, INTA AC1-9, and INTA AC1-14 were tentatively identified as *Enterobacter cloacae*, INTA AC1-4 as *Staphylococcus gallinarum,* and INTA AC1-8 as *S. succinus*. These biochemical assignments should be regarded as preliminary; definitive taxonomic identification of representative isolates was achieved through whole-genome sequencing, as detailed in Section 3.3.

### 3.3. Genome-Resolved Characterization of Selected Representative Culturable Gut Isolates

A total of five representative cultivable gut-associated microorganisms, comprising three bacterial and two yeast isolates, were selected for deep genomic resolution through whole-genome sequencing (WGS). Rather than maximizing taxonomic coverage, isolate selection was intentionally focused on a limited number of morphologically and phenotypically distinct taxa to prioritize in-depth characterization, enabling robust phylogenetic placement and detailed comparative and functional interpretation of representative members of the cultivable larval gut microbiota.

#### 3.3.1. Genome-Based Identification and Phylogenetic Placement of Bacterial Isolates

The metrics summarized in Table S1 (Supplementary Information) indicate high-quality draft genome assemblies for all three bacterial isolates. The genome of strain INTA AN1-1 was the largest (5,080,317 bp) and exhibited high assembly continuity, as reflected by the highest N50 value (290,102 bp), lowest L50 (7), and highest gene content (4908 total predicted genes). In contrast, INTA AC1-4 showed a smaller (2,216,745 bp) and more fragmented assembly, with a higher number of contigs (152) and a lower N50 value (27,591 bp). INTA AC1-8 displayed reduced fragmentation (38 contigs), with a total assembly length of 2,843,947 bp and an N50 of 116,088 bp, although its N50 value was lower than that of INTA AN1-1, indicating differences in assembly structure. GC content differed markedly across isolates, consistent with their taxonomic differences (55.01% in INTA AN1-1 vs. 31.35% and 32.88% in INTA AC1-4 and INTA AC1-8, respectively). Annotation outputs revealed comparable proportions of hypothetical proteins (31.4%, 35.5%, and 32.3% of CDS, respectively) and complete complements of rRNA, tRNA, and tmRNA genes across isolates (ribosomal, transfer, and transfer-messenger RNA, respectively). CheckM analysis confirmed high completeness for all three genomes (99.73%, 99.51%, and 99.45%, respectively), with low estimated contamination (0.19%, 0.17%, and 1.93%, respectively). Consistently high completeness values and low estimated contamination further support the reliability of these draft genomes for downstream phylogenomic and comparative analyses.

**Figure 2:**
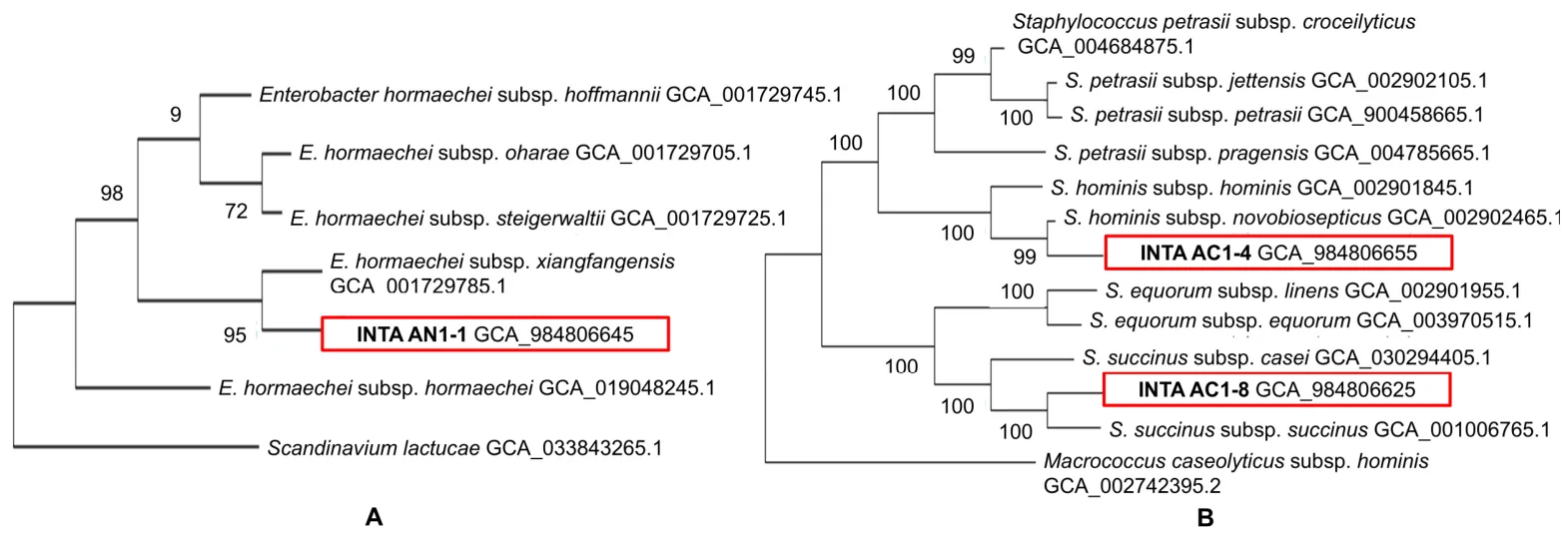
Genome-based phylogenomic identification of bacterial isolates from healthy *Alphitobius diaperinus* larvae. Phylogenomic trees were reconstructed using GToTree for INTA AN1-1 (**A**) and for the *Staphylococcus* isolates INTA AC1-4 and INTA AC1-8 (**B**). Type strain genomes were retrieved from GenBank based on taxonomic information obtained from List of Prokaryotic names with Standing in Nomenclature (LPSN). *Scandinavium lactucae* and *Macrococcus caseolyticus* subsp. *hominis* were included as outgroups in panels A and B, respectively. The trees depict the phylogenetic relationships between each isolate and their closest related type strains. Tree visualization was performed using MEGA. Bootstrap support values (%) are indicated at the nodes.

Whole-genome comparisons against reference type strains resolved the phylogenetic placement of all three bacterial isolates (Figure 2). Isolate INTA AN1-1 was placed closest to the type strain of *E. hormaechei* subsp. *xiangfangensis* (Figure 2A). Isolate INTA AC1-4 was grouped within the *S. hominis* lineage and was placed closest to the type strain of *S. hominis* subsp. *novobiosepticus* (Figure 2B). Similarly, isolate INTA AC1-8 clustered with the reference strain of *S. succinus* subsp. *succinus* (Figure 2B). Overall, phylogenomic placement based on whole-genome data provided robust taxonomic assignments for all three isolates and was consistent with the genome quality metrics reported in Table S1.

Genome-based relatedness metrics summarized in Table 1 confirmed the phylogenomic placements inferred from whole-genome comparisons and excluded the possibility that the analyzed isolates represent novel species or subspecies. Isolate INTA AN1-1 showed the highest genomic similarity to the type strain of *E. hormaechei* subsp. *xiangfangensis*, with dDDH values exceeding the accepted cutoff for species and subspecies delineation, ANI values close to 99%, minimal G+C content differences, and high Tetra z-scores. Comparisons with other *E. hormaechei* subspecies yielded lower dDDH and ANI values, supporting its assignment to the *xiangfangensis* subspecies.

Isolate INTA AC1-4 exhibited the highest dDDH and ANI values relative to *S. hominis* subsp. *novobiosepticus*, together with negligible G+C content differences and high Tetra z-scores, clearly differentiating it from other *Staphylococcus* species included in the analysis.

Similarly, isolate INTA AC1-8 showed maximal genomic relatedness to *S. succinus* subsp. *succinus*, with dDDH and ANI values consistent with species-level assignment and substantially lower similarity to other *Staphylococcus* taxa.

In all cases, ANI values exceeded the 96% threshold, and dDDH values were above 79%, thresholds commonly applied to delimit novel bacterial species and subspecies. Overall, the concordant results obtained from dDDH, ANI, G+C content difference, and Tetra correlation analyses provide robust genome-based evidence supporting the taxonomic identification of all three bacterial isolates.

Comparative genome annotation revealed broadly similar levels of functional annotation across the three gut-associated bacterial isolates (Table S2, Supplementary Information).

eggNOG-mapper: all genomes showed complete assignment to eggNOG orthologs and a high proportion of proteins classified into COG (Clusters of Orthologous Genes) functional categories, with a comparable number of COG categories detected in the three isolates (22, 21, and 22, respectively), indicating well-annotated and functionally resolved genomes. Type VI secretion system components were identified based on eggNOG functional annotations using a keyword-based search for conserved core components. Additional quantitative differences were observed across multiple functional categories. Compared with INTA AC1-4 and INTA AC1-8, isolate INTA AN1-1 encoded higher numbers of transport-related proteins (263 vs. 140 and 186), motility- and chemotaxis-associated functions (27 vs. 0 in both), adhesion- and biofilm-related proteins (48 vs. 2 in both), iron acquisition-associated proteins (24 vs. 2 in both), and type VI secretion system components (24 vs. 3 and 5). Stress response-related protein counts were comparable across the three isolates (21, 20, and 22, respectively).

dbCAN: Clear differences were observed in carbohydrate-active enzyme (CAZyme) repertoires. Isolate INTA AN1-1 encoded a substantially larger number of CAZyme genes and families compared to INTA AC1-4 and INTA AC1-8 (285 genes across 93 families vs. 94 genes/38 families and 147 genes/50 families, respectively), including a higher absolute number and proportion of glycoside hydrolase genes (54.7% vs. 42.6% and 51.7% of CAZyme genes, respectively). INTA AN1-1 also encoded the highest number of auxiliary activity genes (10 vs. 2 and 1) and CBM families (6 vs. 2 in both). In contrast, the two *Staphylococcus* isolates displayed more limited CAZyme complements, whereas INTA AC1-8 consistently showed intermediate values for several of these functional categories. Consistent with these quantitative differences, INTA AN1-1 displayed the broadest functional repertoire among the three bacterial genomes, whereas the two *Staphylococcus* isolates encoded comparatively reduced gene complements across several annotated functional categories.

AntiSMASH: secondary metabolite biosynthetic gene clusters (BGCs) were identified in all three genomes (Table S2, Supplementary Information). INTA AC1-4 contained clusters related to cyclic lactone autoinducers, T3PKS (type III polyketide synthase), nonribosomal siderophores, and terpene precursors. INTA AC1-8 contained clusters associated with terpenes, T3PKS, NRPS (nonribosomal peptide synthetase), hybrid NRPS–terpene systems, terpene precursors, cyclic lactone autoinducers, and nonribosomal siderophores. INTA AN1-1 contained clusters related to NRPS and NRP-metallophores, terpene precursors, nonribosomal siderophores, arylpolyenes, redox cofactor-associated regions, and RiPP-like systems, including azole-containing RiPPs (ribosomally synthesized and post-translationally modified peptides). The total number of predicted BGCs was similar for INTA AN1-1 and INTA AC1-8 (7 each), whereas INTA AC1-4 showed a lower number (4). Notably, no biosynthetic gene clusters associated with known insecticidal or entomopathogenic activities were detected in any of the genomes.

AMRFinderPlus: antimicrobial resistance (AMR) genes were detected at low copy numbers in all three bacterial genomes (3–4 genes per isolate). All identified genes were classified as core (intrinsic) by AMRFinderPlus, and no acquired resistance determinants were detected. *S. hominis* subsp. *novobiosepticus* strain INTA AC1-4 harbored three genes associated with beta-lactams. *S. succinus* subsp. *succinus* strain INTA AC1-8 contained one gene for each of the following classes: rifamycins, lincosamides/pleuromutilins/streptogramins, and beta-lactams. *E. hormaechei* subsp. *xiangfangensis* strain INTA AN1-1 contained one gene for each of the following classes: phenicols, phenicols/quinolones, fosfomycin, and beta-lactams. No acquired polymyxin resistance genes (including *mcr* variants) were detected in any genome.

Manual BLAST: In addition, no acquired polymyxin resistance genes (including *mcr* variants) were detected in any genome. Manual inspection of chromosomal polymyxin-resistance loci in *E. hormaechei* subsp. *xiangfangensis* strain INTA AN1-1 revealed that *pmrB*, *phoQ*, and *mgrB* were identical to the reference sequences, whereas the response regulators *pmrA* and *phoP* showed full-length matches with four amino acid substitutions each but no insertions or deletions.

VFDB (ABRicate): Virulence-associated genes were detected only in INTA AN1-1 (17 hits across 3 functional categories), whereas no VFDB hits were identified in INTA AC1-4 or INTA AC1-8 under the applied thresholds (≥90% identity and ≥90% coverage). In INTA AN1-1, detected genes were mainly associated with the type VI secretion system (T6SS), including core structural components (e.g., *tssB*, *tssC*, and *tssD*/*hcp*), as well as flagellar motility and chemotaxis systems (13 genes), comprising multiple structural and regulatory genes (e.g., *motAB*, *fli* and *flg* operons, and *che* genes). Additionally, a gene encoding a heat-stable enterotoxin (EAST1) was identified.

#### 3.3.2. Genome-Based Identification and Phylogenetic Placement of Yeast Isolates

Genome assembly and annotation statistics for both yeast isolates are summarized in Table S3 (Supplementary Information) and indicate high-quality draft genomes suitable for phylogenomic analyses.

Assembly features: INTA AB1-1 and INTA AB1-4 showed comparable genome sizes (12.41 and 11.72 Mbp, respectively) and GC content (34.92% and 35.60%, respectively), while differences were observed in contiguity metrics. The assembly of INTA AB1-4 exhibited higher continuity, with fewer contigs (152 vs. 184), higher N50 values (286,720 bp vs. 135,982 bp), lower L50 (14 vs. 27), and larger maximum contig size (824,627 bp vs. 540,439 bp). In contrast, INTA AB1-1 showed greater fragmentation, although both genomes displayed features consistent with moderate fragmentation typical of draft fungal genomes, including contigs exceeding 500 kb.

Masked repeats: overall repetitive content was low in both genomes, with transposable elements accounting for only a very small fraction of the assemblies (0.05% in INTA AB1-1 and 0.00% in INTA AB1-4). The repeat landscape was dominated by simple repeats (1.99% and 0.78%, respectively) and low-complexity regions (0.47% and 0.16%, respectively), whereas retrotransposon-related elements—including LINEs (Long Interspersed Nuclear Elements; 0.04% in INTA AB1-1) and DNA transposons (0.01% in INTA AB1-1) were either absent or detected at extremely low proportions. SINEs and LTR elements were absent in both isolates.

Annotation features: genome annotation indicated high completeness for both yeast genomes, with BUSCO scores approaching 99% (98.5% for INTA AB1-1 and 98.8% for INTA AB1-4) and minimal estimated contamination (0.1% and 0.2%, respectively). The total number of predicted genes (5778 and 5815, respectively) and Gene Ontology (GO) terms (1951 and 1963, respectively) were similar between the two isolates, indicating comparable annotation depth and functional coverage. Overall, these results indicate that the draft genome assemblies of INTA AB1-1 and INTA AB1-4 are structurally sound and well annotated, providing a robust foundation for downstream comparative and functional analyses of cultivable gut-associated yeasts.

**Figure 3:**
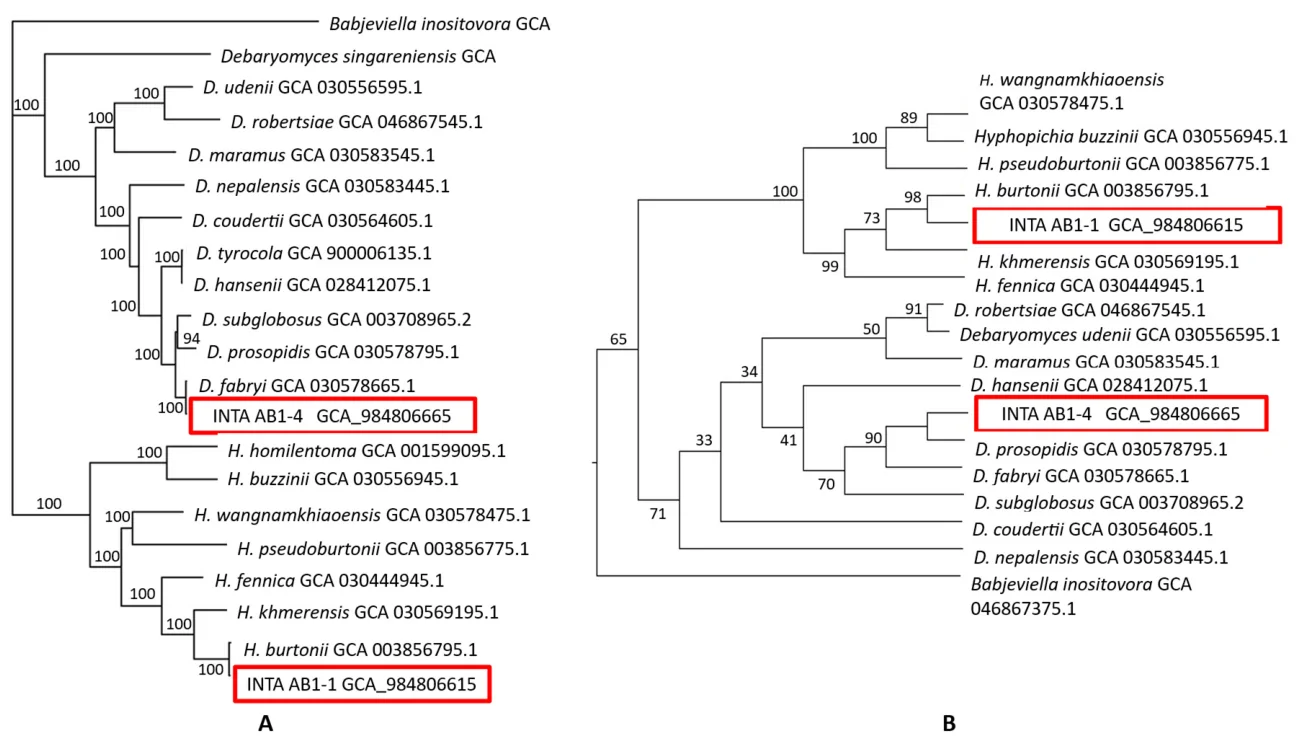
Phylogenetic placement of the yeast isolates INTA AB1-1 and INTA AB1-4. (**A**) Genome-based phylogenomic tree inferred from concatenated single-copy BUSCO orthologs, showing the relationships of the yeast isolates INTA AB1-1 and INTA AB1-4 to closely related type strains. BUSCO analyses were performed using the Debaryomycetaceae_odb12 lineage dataset, and high-confidence shared orthologs were concatenated and aligned at the amino acid level. Phylogenomic inference was conducted using IQ-TREE multicore v2.4.0 with 1000 bootstrap replicates. Bootstrap support values are indicated at the nodes. Branch lengths are proportional to the number of substitutions per site. (**B**) ITS-based phylogenetic tree inferred from the ITS1–NL4 region. The analysis includes reference yeast taxa and the isolates INTA AB1-1 and INTA AB1-4, recovered from the cultivable gut microbiota of *A. diaperinus* larvae. Phylogenetic reconstruction was performed in MEGA using 1000 bootstrap replicates. Bootstrap support values are shown at the nodes and range from 33 to 100. The number of taxa included in panel (**B**) is lower than in panel (**A**), as only genome assemblies meeting a zero-mismatch criterion at both the start and end positions of the ITS1–NL4 region extraction were retained. The scale bar represents branch length in substitutions per site.

Genome-based phylogenomic analysis based on concatenated single-copy BUSCO orthologs resolved the placement of both yeast isolates with high support (Figure 3A). Isolate INTA AB1-1 clustered with the reference strain of *H. burtonii*, forming a well-supported clade within the *Hyphopichia* lineage. Similarly, isolate INTA AB1-4, grouped with the reference genome of *D. fabryi*, was clearly separated from other closely related *Debaryomyces* species included in the analysis.

Regarding the ITS-based phylogenetic analysis shown in Figure 3B, the topology obtained here is based on longer sequences compared to that inferred during the preliminary identification based on partial ITS sequences (see Data Availability Statement). In that study, sequence lengths were limited to 446 bp for INTA AB1-1 and 612 bp for INTA AB1-4. In the present work, the availability of draft genome assemblies enabled the extraction of the complete ITS1–NL4 region, resulting in sequence lengths of 1004 bp for INTA AB1-1 and 1246 bp for INTA AB1-4. Despite the increased sequence length and improved phylogenetic resolution, the resulting ITS-based topology is fully congruent with previous findings, supporting the same taxonomic placement of both isolates. Thus, the current analysis provides a more comprehensive ITS framework while reinforcing the conclusions derived from earlier partial-sequence data.

Pairwise nucleotide and amino acid identity metrics, summarized in Table 2, provided quantitative support for the taxonomic placement of the two yeast isolates. For each yeast isolate (INTA AB1-1 and INTA AB1-4), pairwise comparisons were performed against the three most closely related type strains associated with each yeast recovered from the gut of *A. diaperinus*, respectively.

For INTA AB1-1, the highest sequence identity values were obtained in comparison with the type strain of *H. burtonii*, including high nucleotide identity across the complete ITS1–NL4 region and very high amino acid identity based on conserved BUSCO protein sequences. Genome-wide nucleotide identity calculated from concatenated BUSCO coding sequences also exceeded values observed in comparisons with the other closely related *Hyphopichia* type strains, which showed lower identity scores and higher numbers of divergent nucleotide and amino acid positions.

Similarly, INTA AB1-4 exhibited maximal nucleotide and amino acid identity values when compared with the type strain of *D. fabryi*. Both ITS-based and genome-scale comparisons showed near-complete identity, with minimal numbers of divergent sites, whereas comparisons with other closely related *Debaryomyces* type strains resulted in lower identity values and increased sequence divergence.

To assess whether either isolate could represent a novel species, sequence identity values were interpreted using established reference thresholds. First, average amino acid identity (AAI) values were not within the range expected for interspecific comparisons, as AAI values ≤99% have been proposed as indicative of distinct yeast species when comparing isolates [45]. Second, MAOG (Multiple Alignment of Orthologous Genes)–based amino acid identity values were above the >95% species-level cutoff applied within *Debaryomyces* [46]. Finally, nucleotide identity values obtained from the complete ITS1–NL4 rDNA region, encompassing both the ITS region and the LSU D1/D2 domains, were consistent with yeast DNA barcoding thresholds reported by Vu et al. [47] (98.41% for ITS and 99.51% for LSU). Collectively, these genome- and marker-based metrics exclude the possibility that INTA AB1-1 or INTA AB1-4 represent novel yeast species.

Overall, the concordant results obtained from ITS-based nucleotide identity, core genome nucleotide identity, and MAOG/AAI amino acid identity analyses provide robust genome- and sequence-level evidence confirming that both yeast isolates correspond to previously described species within the genera *Hyphopichia* and *Debaryomyces*.

A detailed description of the results summarized in Table S4 (see Supplementary Information) is provided below.

InterProScan: Functional annotation indicated a high degree of protein characterization in both yeast isolates. InterProScan analysis assigned at least one InterPro entry to 91.58% of predicted proteins in INTA AB1-1 and 98.18% in INTA AB1-4, with Pfam domains detected in 86.46% and 92.47% of proteins, respectively. The number of unique InterPro entries and Pfam domains was comparable between isolates, with slightly higher values observed for INTA AB1-4. The number of unique InterPro entries was comparable between isolates (6780 in INTA AB1-1 and 6745 in INTA AB1-4), as were unique Pfam domains (3283 and 3328, respectively), with slightly higher values observed for INTA AB1-4.

Transporters: To avoid false positives, only proteins annotated with the GO term “transmembrane transporter activity” (GO:0022857) and containing explicit transporter-related descriptors were retained. A strict filtering strategy identified a substantial repertoire of bona fide transmembrane transporters in both yeast isolates (73 in INTA AB1-1 and 96 in INTA AB1-4). Major facilitator superfamily (MFS) transporters (36 and 63, respectively) and sugar transporters (30 and 48, respectively) predominated, particularly in INTA AB1-4, consistent with a metabolically versatile, gut-associated lifestyle. Amino acid/peptide transporters were detected in comparable numbers in both isolates (16 in INTA AB1-1 and 13 in INTA AB1-4). INTA AB1-4 exhibited an expanded transporter repertoire compared to INTA AB1-1, in agreement with its broader CAZyme profile. No *bona fide* ABC-type transmembrane transporters were detected in either genome under the applied filtering criteria.

Curated gut-related functional categories (InterProScan-derived; this study): In addition to core metabolic and transport functions, both yeast genomes encoded a substantial repertoire of proteins associated with gut stress and tolerance-related functions. A total of 456 and 358 unique stress-associated proteins were identified in INTA AB1-1 and INTA AB1-4, respectively. Among these, 36 and 28 proteins were assigned to two or more stress-related functional categories, respectively. Oxidative stress response was the predominant stress-related category in both isolates. Both yeast isolates encoded a comparable number of proteins associated with microbial interaction-related functions, with 88 proteins identified in INTA AB1-1 and 87 in INTA AB1-4. In both genomes, adhesion-, lectin-, and flocculin-related proteins represented the most abundant category, accounting for 62.5% and 66.67% of the total, respectively. Proteins associated with cell wall or surface anchoring represented 23.9% in INTA AB1-1 and 17.24% in INTA AB1-4, while the remaining fraction corresponded to other interaction-related categories. Cell wall or surface anchoring-associated proteins represented 23.9% (21 proteins) in INTA AB1-1 and 17.24% (15 proteins) in INTA AB1-4, while the remaining fraction (13.6% and 16.09%, respectively) corresponded to other interaction-related categories.

OrthoVenn3: Orthology analysis revealed a highly conserved gene repertoire between the two yeast isolates. A total of 5544 and 5568 orthologous clusters were inferred for INTA AB1-1 and INTA AB1-4, respectively, with the vast majority of clusters shared between isolates (5526 in AB1-1 and 5553 in AB1-4). Only a small number of isolate-specific clusters was detected, comprising 10 unique clusters in INTA AB1-1 and 9 in INTA AB1-4. In addition, 209 singleton proteins were identified in INTA AB1-1 and 162 in INTA AB1-4. The total number of orthologous clusters corresponds to the number of orthogroups inferred in each pairwise OrthoVenn3 run.

dbCAN: CAZyme profiling revealed a broad and balanced repertoire of carbohydrate-active enzymes in both yeast isolates. A total of 721 and 753 CAZyme-encoding genes were identified in INTA AB1-1 and INTA AB1-4, respectively, spanning approximately 140 CAZyme families (140 and 142, respectively). Glycoside hydrolases accounted for nearly half of the predicted CAZymes (48.96% and 49.4%, respectively), while auxiliary activity enzymes (55 and 57 genes, respectively) and carbohydrate-binding modules (12 CBM families in both isolates) were present at moderate and comparable levels in both genomes. Overall, the CAZyme profiles were highly similar between isolates and consistent with a non-pathogenic, gut-associated lifestyle.

FungiSMASH: Secondary metabolite biosynthetic gene clusters (BGCs) were identified in the yeast genomes. Both INTA AB1-1 and INTA AB1-4 contained five clusters, all associated with terpene biosynthesis. In each strain, three clusters corresponded to terpene regions and two to terpene precursor regions. All predicted clusters corresponded to terpene or terpene-precursor pathways, and none showed similarity to gene clusters associated with known insecticidal or entomopathogenic activities.

## 4. Discussion

### 4.1. Gut-Associated Microorganisms in Insect Larvae: Possible Ecological Relevance and Biotechnological Perspectives

As mentioned in the Introduction of this work, gut-associated microbial communities in insects have ecological relevance and biotechnological potential, but bacterial communities have been more extensively characterized than intestinal yeasts. While some insect species harbor stable and functionally specialized gut communities, most insect guts contain relatively few microbial species, and in many cases are only opportunistically and sparsely colonized by bacteria commonly found in other environments [48]. Many questions remain unanswered, including whether specific intestinal factors select for particular yeast taxa, the extent to which host immunity shapes these associations, and what benefits yeasts themselves derive from living within the insect gut. Genome-based annotation provides additional tools to address some of these questions and allows prediction of functional and metabolic potential.

### 4.2. Validation of Taxonomic Identification Against Previous A. diaperinus Microbiota Findings

While previous studies on the gut microbiota of *A. diaperinus* have been noted in the Introduction, one study deserves particular attention in interpreting our results. It is of interest to compare our findings with the master’s thesis by Torres Valderrama 2022 [8], as four of the five microorganisms identified in our study overlap with those reported in her work. Using a metagenomic approach, she identified members of the genera *Enterobacter* and *Staphylococcus*, as well as *H. burtonii*, from the microbiome of *A. diaperinus* collected at a poultry farm.

### 4.3. From Taxonomic Profiling to Genome-Resolved Ecological Insights

Despite these previous reports describing the gut-associated microbiota of *A. diaperinus*, the present study provides a novel contribution by moving beyond taxonomic profiling studies to genome-resolved characterization. Here, we sequenced and analyzed five draft genomes derived from aerobically cultivable gut-associated microorganisms, including three bacterial isolates and two yeast isolates, selected following a robust preliminary molecular identification based on 16S rRNA gene sequencing for bacteria and ITS region analysis for yeasts. All isolates originated from a long-term, stable laboratory colony maintained for several years on a commercial chick starter feed (Ganave), designed to simulate broiler house nutritional conditions while preserving insect health under controlled and sanitary laboratory conditions.

As a result of the present work, the morphotype most frequently recovered from a pooled homogenate of the cultivable gut microbiota of *A. diaperinus* under the specific cultivation conditions employed was taxonomically identified as *E. hormaechei* subsp. *xiangfangensis* strain INTA AN1-1. This taxon was originally described as *Enterobacter xiangfangensis*, first isolated from Chinese traditional sourdough by Gu et al., 2014 [49]. Subsequent comparative genomic analyses led to its reclassification as *E. hormaechei* subsp. *xiangfangensis*, as proposed by Sutton et al., 2018 [50].

Regarding the staphylococcal isolates recovered from the cultivable gut microbiota of *A. diaperinus*, *S. hominis* subsp. *novobiosepticus* was originally described by Kloos et al., 1998 [51] following its isolation from human blood cultures. In contrast, *Staphylococcus succinus* was described by Lambert et al.,1998 [52] based on isolates recovered from Dominican amber, highlighting its association with ancient resin environments; the subspecies designation *succinus* was subsequently established upon proposal of *S. succinus* subsp. *casei* [53].

Among the yeast isolates recovered from the cultivable gut microbiota, strain INTA AB1-1 was taxonomically identified as *H. burtonii*. The species was originally described as *Pichia burtonii* by Boidin et al., 1964 [54], later reassigned to the genus *Hyphopichia* by von Arx and van der Walt 1987 [55], and the type strain was originally isolated from pollen in Brazil, as documented in international culture collections. The INTA AB1-4 strain was taxonomically identified in the present study as *D. fabryi*. The species was originally described by Ota in 1924 [56]. A comprehensive modern taxonomic reassessment of *D. fabryi* was later provided by Groenewald et al., 2008 [57].

Although none of the five microorganisms identified at the species or subspecies level by whole-genome sequencing in our study represent novel bacterial or yeast taxa, their recovery from the insect gut is noteworthy, as none of the original species’ descriptions were linked to an insect intestinal microbiome. This observation highlights the ecological versatility of these microorganisms.

### 4.4. Ecological Implications of Genome Annotation in Gut-Associated Bacterial Isolates

In the initial stage of this work, bacterial isolates were identified to the genus level based on the partial 16S rRNA gene, and their relative abundances were assessed. In the second stage, representative isolates were further characterized by Illumina-based whole-genome sequencing, enabling taxonomic resolution at the species and subspecies levels. This genome-resolved approach may allow the inference of functional traits potentially relevant to microbial ecology within the larval gut, providing a refined framework to interpret host–microbe interactions under controlled rearing conditions.

Comparative genome annotation suggested marked differences in the predicted functional repertoires of the three gut-associated bacterial isolates (Table S2, see Supplementary Information). Although all genomes showed high annotation coverage and a comparable distribution of core COG functional categories, *E. hormaechei* subsp. *xiangfangensis* strain INTA AN1-1—identified as the most frequently recovered member of the cultivable microbiota of healthy *A. diaperinus* larvae maintained under stable laboratory rearing conditions and fed a standard chick starter diet —consistently encoded the largest number of transport-related proteins, type VI secretion system components, motility- and chemotaxis-associated functions, adhesion- and biofilm-related proteins, iron acquisition systems, and carbohydrate-active enzymes, together with the highest diversity of CAZyme families and auxiliary activities.

Notably, INTA AN1-1 displayed the broadest predicted functional profile among the three isolates, encoding multiple genomic traits potentially associated with gut colonization and ecological persistence. This profile is consistent with efficient nutrient acquisition, carbohydrate processing, and dynamic interaction with the gut environment. The expanded CAZyme repertoire detected in INTA AN1-1 is particularly relevant in the context of typical chick starter feed formulations. Such feeds are nutritionally complex, rich in starch, non-starch polysaccharides, and plant-derived fibers, substrates that require enzymatic degradation prior to assimilation. The diversity of glycoside hydrolases, carbohydrate-binding modules, and auxiliary activities encoded in this strain may align with the range of substrates typically present in such formulations, potentially enabling the degradation of complex feed components within the larval gut and contributing to nutrient liberation for the host.

It should be noted that the genome of INTA AN1-1 also encoded genes associated with type VI secretion systems, additional virulence-associated factors, and the EAST1 enterotoxin, traits that, in the clinical literature, are often discussed in connection with the opportunistic pathogenic potential of *E. hormaechei* in immunocompromised human hosts. However, members of the genus *Enterobacter* can be isolated from insect-associated microbiota, plants, soil, and other natural environments, where they participate in non-pathogenic ecological interactions with their hosts [58,59,60]. Many genes annotated as “virulence factors,” including components of type VI secretion systems and toxin-like determinants such as EAST1, are increasingly recognized to play broader roles in interbacterial competition, niche colonization, and environmental persistence, rather than necessarily indicating pathogenicity toward the insect host. Their detection in INTA AN1-1 should therefore be interpreted with caution and does not, by itself, imply a pathogenic relationship with *A. diaperinus* under the rearing conditions evaluated in this study.

Unlike INTA AN1-1, the staphylococcal isolates *S. hominis* subsp. *novobiosepticus* strain INTA AC1-4 and *S. succinus* subsp. *succinus* strain INTA AC1-8 encoded comparatively reduced repertoires of transport, colonization-associated, and carbohydrate-active functions, with INTA AC1-8 displaying an intermediate functional profile. Although their capacity for complex carbohydrate processing appears limited relative to INTA AN1-1, their presence within the cultivable microbiota may point to a complementary, though more limited, role in gut digestive processes.

Despite these quantitative differences, all three genomes encoded stress response-associated proteins and biosynthetic gene clusters, whereas no biosynthetic clusters associated with known insecticidal or entomopathogenic activity and no acquired antimicrobial resistance determinants were detected. Collectively, these findings suggest functionally differentiated but potentially complementary genomic repertoires among the cultivable bacterial members of the larval gut microbiota.

### 4.5. Ecological Implications of Genome Annotation in Gut-Associated Yeast Isolates

While ITS-based identification provides an initial taxonomic assignment, its resolution is limited compared to that of genome-based approaches. In contrast, average nucleotide identity (ANI) derived from whole-genome sequencing is currently considered one of the gold standards for species-level delineation in prokaryotes, and is increasingly recognized as a reliable approach for yeast species identification, enabling more accurate and robust taxonomic classification of microbial isolates [43,46]. This genome-based resolution provides a useful framework for interpreting the predicted functions and possible ecological roles of the isolates.

Comparative functional annotation of the two yeast genomes revealed a high degree of similarity across multiple annotation layers, including InterPro, Pfam, CAZyme repertoires, transporter profiles, and stress-associated functional categories (Table S4, see Supplementary Information). This overall functional concordance suggests that INTA AB1-1 and INTA AB1-4 share broadly comparable ecological strategies within the gut environment of *A. diaperinus* larvae under laboratory rearing conditions.

Both yeast genomes encoded extensive repertoires of carbohydrate-active enzymes, with glycoside hydrolases representing approximately half of the detected CAZyme genes, together with auxiliary activities, carbohydrate-binding modules, and a substantial number of sugar transporters—a profile suggesting substantial potential for carbohydrate utilization that mirrors the dietary-substrate adaptation inferred for the bacterial isolates (see Section 4.4). This suggests that, alongside *E. hormaechei* subsp. *xiangfangensis* strain INTA AN1-1, these fungi may act as complementary carbohydrate-degrading partners within the cultivable gut community.

Importantly, neither yeast genome harbored biosynthetic gene clusters associated with known insecticidal or entomopathogenic activity, nor did they display genomic features typically linked to pathogenic or invasive lifestyles. Instead, proteins associated with microbial interaction were predominantly related to adhesion, lectin- or flocculin-like functions, and cell wall or surface anchoring—in yeasts, commonly mediated by cell wall–anchored adhesins such as the *Flo* family, whose presence and architecture vary widely across taxa [61]—consistent with substrate attachment, biofilm-like associations, or persistence within the gut lumen.

Notably, both yeast isolates were recovered on *B. cereus* agar, a polymyxin B–supplemented selective and differential medium that is typically inhibitory to many microorganisms. Their ability to grow under these conditions suggests tolerance to the selective pressure imposed by the medium. This observation indicates that these isolates were capable of growing on the cultivation medium employed in our protocol, although it does not by itself establish their abundance or colonization status in vivo.

*H. burtonii* has been reported as an osmotolerant and halotolerant yeast [62] frequently associated with sugar-rich food matrices such as bakery products, where it has been identified in previous studies among the “chalk yeasts” responsible for spoilage [63]. In this context, *H. burtonii* strain INTA AB1-1, for which approximately 90 interaction-associated proteins were detected, displays a repertoire consistent with the potential for surface attachment and temporary persistence without indicating specialization toward host invasion or pathogenicity.

*D. fabryi* strain INTA AB1-4 is phylogenetically close to the *D. hansenii* clade [64], and within the genus, tolerance to high salinity and osmotic challenge has been documented in the literature under laboratory conditions [65]. In yeasts, adaptation to physicochemical stresses is known to involve extensive remodeling of the cell wall and its associated proteins [66], and previous studies have shown that *D. fabryi* forms robust biofilms in seawater-based media [67], supporting a lifestyle capable of stable associations in saline environments.

In the present study, stress-related functional categories were also highly conserved between the two isolates, with oxidative stress response emerging as the most frequently represented category. This pattern may suggest adaptation to physicochemical conditions within the insect gut, including redox fluctuations and exposure to reactive oxygen species generated during digestion or microbial competition.

Taken together, the functional profiles of INTA AB1-1 and INTA AB1-4 are consistent with their potential occurrence as members of the cultivable gut-associated mycobiota of *A. diaperinus* larvae. Their genomic features support a non-pathogenic, metabolically versatile, and primarily substrate-associated lifestyle, potentially contributing to nutrient processing within the gut ecosystem under the laboratory conditions examined.

### 4.6. Limitations of the Present Study

Several limitations should be acknowledged. First, the study focused exclusively on the aerobic culturable fraction of the gut microbiota and therefore represents only a subset of the total microbial diversity present in the larval gut of *A. diaperinus*. Second, isolates were obtained from a laboratory colony maintained under controlled rearing conditions since 2012 without the introduction of new external specimens and may not fully reflect the microbial composition of field populations. Third, isolates were obtained from a single pooled sample of fifteen larvae maintained in fasting conditions for 24 h prior to dissection, which precludes assessment of inter-individual variation, prevalence of individual taxa, stability of microbial associations, and statistically supported comparisons among culture media. Fourth, no colonization or persistence assays have yet been performed. Fifth, genome assemblies were generated using short-read Illumina sequencing, resulting in draft-quality genomes of high quality and completeness that do not permit complete genome closure. Finally, functional inferences were based on genome annotation, representing an initial characterization with additional functional potential awaiting further exploration. The predicted traits discussed herein should therefore be interpreted as hypotheses to be addressed in future studies rather than demonstrated biological functions.

## 5. Conclusions

If we revisit the hypotheses formulated in the introduction, our results validate all three proposed hypotheses.

First hypothesis—whole-genome sequencing enables reliable taxonomic identification at species/subspecies level: we isolated and characterized five culturable microorganisms from a single pooled sample of fifteen larvae from the gut of *A. diaperinus* reared under laboratory conditions. Strains INTA AN1-1, INTA AC1-4, INTA AC1-8, INTA AB1-1, and INTA AB1-4 were identified as *E. hormaechei* subsp. *xiangfangensis*, *S. hominis* subsp. *novobiosepticus*, *S. succinus* subsp. *succinus*, *H. burtonii*, and *D. fabryi*, respectively. While 16S/ITS allowed only genus-level identification, WGS achieved species/subspecies resolution. Although none represent a novel taxon, not a single isolate was originally described from the insect gut microbiota, demonstrating the ecological versatility of these microorganisms. Our work provides the first draft genomic description of aerobically cultivable gut microbiota of *A. diaperinus* larvae under laboratory conditions. Despite this, the cultivable fraction inevitably represents only a minor proportion of the total gut community; this methodological choice confers a decisive practical advantage: the recovery of viable isolates enables direct functional experimentation.

Second and third hypotheses—polyphasic approach validates phenotypic-genomic congruence; genome annotation reveals comprehensive functional potential: *E. hormaechei* subsp. *xiangfangensis* strain INTA AN1-1 was the most frequently recovered component under the experimental conditions employed, displaying broad metabolic versatility, extensive carbohydrate-active enzyme repertoires, and transporter systems that potentially facilitate nutrient processing within the intestinal environment, based on genomic predictions from our in silico analyses. In contrast, the two staphylococcal isolates exhibited reduced metabolic complexity and limited carbohydrate-degrading capacity, which may reflect a more limited, though not necessarily less relevant, ecological role within the gut environment. Yeast isolates displayed functional potential for carbohydrate utilization, surface-associated interaction traits, and stress tolerance. Their unexpected recovery from *B. cereus*-selective media (Polymyxin B-supplemented) suggests tolerance to selective pressure. This genomic characterization of the aerobically culturable bacteria and yeasts isolated from pooled larval midguts of *A. diaperinus* establishes a critical foundation for future experimental validation of these gut-associated microorganisms and their ecological significance within the lesser mealworm.

## Figures and Tables

**Table 1:** Type strains most closely related to INTA AN1-1, INTA AC1-4, and INTA AC1-8, respectively, showing digital DNA–DNA hybridization (dDDH) values, percent G+C content difference, Average Nucleotide Identity (ANI), and Tetra z-score, as estimated using the TYGS pipeline.

Reference Strain	GenBank Assembly Accession	dDDH (d0, %)	dDDH (d4, %)	dDDH (d6, %)	G+C Content Difference (%)	ANIb [%]	ANIm [%]	Tetra z-Score
*Enterobacter hormaechei* subsp. *xiangfangensis* LMG 27195	GCF_001729785	83.8	93.0	88.2	0.27	98.84	99.19	0.99914
*Enterobacter hormaechei* subsp. *oharae* DSM 16687	GCF_001729705	78.5	76.1	81.0	0.57	96.99	97.29	0.99875
*Enterobacter hormaechei* subsp. *steigerwaltii* DSM 16691	GCF_001729725	75.5	75.7	78.3	0.55	96.70	97.24	0.99877
*Staphylococcus hominis* subsp. *novobiosepticus* CCUG 42399	GCF_002902465	84.7	92.7	88.9	0.04	98.91	99.17	0.9986
*Staphylococcus hominis* subsp. *hominis* NCTC 11320	GCF_002901845	88.4	79.8	89.7	0.03	97.46	97.75	0.99941
*Staphylococcus petrasii* subsp. *pragensis* CCM 8529	GCA_004785665	33.6	22.7	29.8	1.72	79.64	85.55	0.9652
*Staphylococcus succinus* subsp. *succinus* DSM 14617	GCA_001006765	91.9	82.2	92.9	0.06	97.73	97.96	0.99912
*Staphylococcus succinus* subsp. *casei* DSM 15096	GCF_030294405.1	87.4	65.0	86.1	0.16	95.61	95.77	0.99794
*Staphylococcus equorum* subsp. *equorum* NCTC 12414	GCA_900458565	33.1	23.3	29.6	0.23	79.79	84.34	0.99068

**Table 2:** Pairwise nucleotide and amino acid identity metrics between the yeast isolates INTA AB1-1 and INTA AB1-4 and their closest related type strains. Nucleotide identity values were calculated for the ITS1–NL4 region and, independently, at the genome level using concatenated single-copy BUSCO coding sequences. Genome-wide nucleotide identity based on BUSCO orthologs reflects similarity across the conserved core genome and differs from FastANI estimates, which were computed from whole-genome assemblies in the Galaxy platform. MAOG-based amino acid identity values were estimated from a MAFFT alignment of concatenated conserved BUSCO protein sequences. The number of divergent nucleotides and amino acid positions was inferred from the corresponding identity metrics. Together, these results support the assignment of INTA AB1-1 and INTA AB1-4 to previously described species within the genera *Hyphopichia* and *Debaryomyces*, respectively.

Isolate	Reference Material Information		ITS-Based Metrics		Core Proteome Metrics		Whole-Genome Metrics		
	Strain	GenBank Assembly Accession	ITS1–NL4 Nucleotide Identity (%)	Number of Divergent Nucleotides (ITS1–NL4)	MAOG-Based Amino Acid Identity (%)	Number of Divergent Amino Acid Positions	FastANI (%)	Genome-Wide Nucleotide Identity (%)	Number of Divergent Nucleotides (Genome-Wide, bp)
INTA AB1-1	*Hyphopichia burtonii makgeolli*	GCA_003856795.1	98.89	11	99.86	1655	98.38	99.41	17,701
	*Hyphopichia khmerensis CBS 9784*	GCA_030569195.1	95.54	44	98.37	19,840	81.84	83.98	468,104
	*Hyphopichia fennica NRRL Y-7505*	GCA_030444945.1	93.18	66	97.00	36,424	80.20	82.13	477,416
INTA AB1-4	*Debaryomyces fabryi* NRRL Y-17914	GCA_030578665.1	100	0	99.998	33	99.47	99.64	14,537
	*Debaryomyces subglobosus* NRRL Y-6666	GCA_003708965.2	99.92	1	99.63	5102	90.17	92.02	309,609
	*Debaryomyces prosopidis NRRL Y-27369*	GCA_030578795.1	100	0	99.23	10,497	87.11	89.36	411,795

## Data Availability

Nucleotide sequences of the 16S rRNA gene for the ten bacterial isolates (INTA AN1-1, AN1-5, AN1-10, AN1-15, AC1-3, AC1-6, AC1-9, AC1-14, AC1-4, and AC1-8) and partial ITS region sequences for the two yeast isolates (INTA AB1-1 and AB1-4) have been deposited in GenBank under accession numbers OP339834.1, OP346784.1, OP346981.1, OP347118.1, OP348220.1, OP348874.1, OP348886.1, OP351273.1, OP348929.1, OP348932.1, OP348991.1, and OP348992.1, respectively. Draft genome sequences for the five isolates (*Enterobacter hormaechei* subsp. *xiangfangensis* strain INTA AN1-1, *Staphylococcus hominis* subsp. *novobiosepticus* strain INTA AC1-4, *Staphylococcus succinus* subsp. *succinus* strain INTA AC1-8, *Hyphopichia burtonii* strain INTA AB1-1, and *Debaryomyces fabryi* strain INTA AB1-4), raw sequencing reads, and partial 18S–ITS1–5.8S–ITS2–partial 28S rRNA gene sequences for the two yeast isolates have been deposited in ENA under BioProject PRJEB114349 (“Draft genome sequences of cultivable bacteria and yeasts from healthy *Alphitobius diaperinus* larvae”). Assembly accessions are GCA_984806645, GCA_984806655, GCA_984806625, GCA_984806615, and GCA_984806665; ITS accessions are OZ482580 (*H. burtonii* strain INTA AB1-1) and OZ482581 (*D. fabryi* strain INTA AB1-4); raw read run accessions are ERR17424246, ERR17424248, ERR17424249, ERR17424250, and ERR17424251. Additional data are included in the article and its Supplementary Information.

## Supplementary Materials

The following supporting information can be downloaded at: https://www.mdpi.com/article/10.3390/insects17080800/s1. Table S1: Assembly, quality, and annotation metrics of the draft genomes of strains INTA AN1-1, INTA AC1-4, and INTA AC1-8; Table S2: Functional genomic features inferred from comparative genome annotation of three gut-associated bacterial isolates; Table S3: Genome assembly and annotation statistics for the yeast isolates INTA AB1-1 and INTA AB1-4; Table S4: Functional genomic annotation summary of the yeast isolates INTA AB1-1 and INTA AB1-4. Additional supplementary scripts for genome annotation parsing and comparative analysis are provided: evaluate.py, buscolist.py, deletegenes.sh, remove_first_line.sh, concatenate_and_add_header.sh, ABRicate_VFDB_Workflow_Supplementary.txt, AMR_Workflow_Supplementary.txt, CAZyme Annotation Using dbCAN.txt, Identification and Classification of Gut Stress-Associated Proteins in Yeast Isolates.txt, Identification and Classification of Microbial Interaction-Associated Proteins.txt, and transporters.py.

## Abbreviations

The following abbreviations are used in this manuscript:

DNA	Deoxyribonucleic Acid
WGS	Whole-Genome Sequencing
NA	Nutrient Agar
BHI	Brain Heart Infusion Agar
BC	*Bacillus cereus* Agar
CFU	Colony-Forming Unit
SEM	Scanning Electron Microscopy
ITS	Internal Transcribed Spacer
16S rRNA	16S Ribosomal Ribonucleic Acid
GC content	Guanine–Cytosine Content
N50, N90	Contig Length Thresholds at which 50% and 90% of the Total Assembly Length Are Covered, Respectively
L50, L90	Minimum Number of Contigs Covering 50% and 90% of the Total Assembly Length, Respectively
WSL	Windows Subsystem for Linux
CDS	Coding Sequence
TYGS	Type Strain Genome Server
ANI	Average Nucleotide Identity
dDDH	digital DNA–DNA Hybridization
ANIb, ANIm	Average Nucleotide Identity calculated using BLAST and MUMmer, respectively
LPSN	List of Prokaryotic Names with Standing in Nomenclature
MEGA	Molecular Evolutionary Genetics Analysis
CAZymes	Carbohydrate-Active enZymes
dbCAN	database for Carbohydrate-active enzyme Annotation
antiSMASH	antibiotics and Secondary Metabolite Analysis SHell
AMR	Antimicrobial Resistance
BLAST	Basic Local Alignment Search Tool
BLASTN	Basic Local Alignment Search Tool Nucleotide
VFDB	Virulence Factor DataBase
GO	Gene Ontology
MFS	Major Facilitator Superfamily
ABC	ATP-Binding Cassette
BUSCO	Benchmarking Universal Single-Copy Orthologs
MAFFT	Multiple Alignment using Fast Fourier Transform
MAOG	Multiple Alignment of Orthologous Genes
rRNA, tRNA, tmRNA	Ribosomal, Transfer, and Transfer-Messenger RNA
BGCs	Biosynthetic Gene Clusters
T3PKS	Type III Polyketide Synthase
NRPS	Non-Ribosomal Peptide Synthetase
RiPPs	Ribosomally Synthesized and Post-Translationally Modified Peptide
LINEs	Long Interspersed Nuclear Elements
AAI	Average Amino Acid Identity
LSU	Large Subunit RNA
COGs	Clusters of Orthologous Genes
