# Supplementary material for: Genomic Characterization of Aerobically Culturable Gut-Associated Bacteria and Yeasts Isolated from Pooled Larval Midguts of the Lesser Mealworm Alphitobius diaperinus (Coleoptera: Tenebrionidae)
Source: Insects. 2026 Aug 2;17(8):800. doi: 10.3390/insects17080800 (PMC13513466; doi:10.3390/insects17080800)
Supplement: Supplementary file 1 [file insects-17-00800-s001.zip › Supplementary Materials 01-08-2026.pdf]

Supplementary Information

Table S1. Assembly, quality, and annotation metrics of the draft genomes of strains INTA AN1-1, INTA AC1-4, and INTA AC1-8. Genome assemblies were evaluated using QUAST v4.4 to obtain standard assembly statistics, including the total number of contigs, largest contig size, total assembly length, GC content, and N50/N75/L50/L75 metrics. Genome completeness and estimated contamination were assessed using CheckM v1.0.18. Genome annotation statistics, including the number of protein-coding genes, annotated CDS, hypothetical proteins, and RNA genes, were obtained from Prokka annotation outputs. All assemblies showed high completeness and low levels of contamination, supporting their suitability for downstream genome-based phylogenomic and comparative analyses.

Genome metrics	INTA AN1-1	INTA AC1-4	INTA AC1-8
**QUAST Assembly metrics**			
Total number of contigs	51	152	38
Largest contig (number of nucleotides)	444584	112787	366305
Total assembly length (nucleotides)	5080317	2216745	2843947
GC content (%)	55.01	31.35	32.88
N50 (bp)	290102	27591	116088
N90 (bp)	63137	7905	36142
L50 value	7	27	7
L90 value	21	84	21
**Genome quality (CheckM)**			
Assembly completeness (%)	99.73	99.51	99.45
Estimated contamination (%)	0.19	0.17	1.93
**Genome annotation (Prokka)**			
Protein-coding genes (CDS)	4853	2155	2667
Annotated CDS (non-hypothetical)	3328	1390	1806
Hypothetical proteins, n (% of CDS)	1525 (31.4%)	765 (35.5%)	861 (32.3%)
tRNA genes	50	39	22
rRNA genes	4	3	4
tmRNA genes	1	1	1
Total predicted genes	4908	2198	2694

Table S2. Functional genomic features inferred from comparative genome annotation of three gut-associated bacterial isolates. These features were inferred using comparative annotation of the three bacterial isolates with eggNOG-mapper (COG assignment), antiSMASH (biosynthetic gene clusters), dbCAN (CAZyme annotation), AMRFinderPlus (antimicrobial resistance–associated genes), and ABRicate with the VFDB database (virulence-associated genes). Antimicrobial resistance–associated genes were classified as core or acquired according to AMRFinderPlus annotations. Chromosomal polymyxin-resistance loci (pmrAB, phoPQ, mgrB) were manually inspected by BLAST against the reference genome of *Enterobacter hormaechei* subsp. *xiangfangensis*, and loci identical to the reference and regulators with amino-acid substitutions are reported separately. VFDB hits were identified using ≥90% sequence identity and ≥90% coverage thresholds. Zeros indicate that no features meeting these criteria were detected.

**Feature**	**INTA AN1-1**	**INTA AC1-4**	**INTA AC1-8**	**Tool / Database**
Proteins assigned to eggNOG orthologs (%)	100.0	100.0	100.0	eggNOG-mapper
Proteins assigned to COG categories (%)	94.5	91.7	93.9	
COG functional categories detected (n)	22	21	22	
Type VI secretion system components (n)	24	3	5	
Transport-related proteins (n)	263	140	186	
Motility / chemotaxis-related proteins (n)	27	0	0	
Adhesion / biofilm-related proteins (n)	48	2	2	
Iron acquisition / siderophore-related proteins (n)	24	2	2	
Stress response-related proteins (n)	21	20	22	
CAZyme genes (total)	285	94	147	CAZymes (dbCAN)
CAZyme families detected (n)	93	38	50	
GH genes (% of CAZyme genes)	54.7	42.6	51.7	
Auxiliary activities genes (n)	10	2	1	
CBM families detected (n)	6	2	2	
Biosynthetic gene clusters (total)	7	4	7	antiSMASH

Biosynthetic gene clusters associated with known insecticidal or entomopathogenic activity (n)	0	0	0	
Antimicrobial resistance–associated genes (n)	4	3	3	AMRFinderPlus
Core / acquired AMR-associated genes (n)	4 / 0	3 / 0	3 / 0	
AMR classes detected (n)	4	1	3	
Acquired polymyxin resistance genes (mcr)	0	0	0	
Chromosomal polymyxin loci identical to reference	pmrB, phoQ, mgrB	NA	NA	Manual BLAST
Chromosomal polymyxin regulators with substitutions	pmrA (4 aa), phoP (4 aa)	NA	NA	
Virulence-associated genes (VFDB hits, n)	17	0	0	
VFDB functional categories detected (n)	3	0	0	VFDB (ABRicate)
Motility/chemotaxis-associated genes (n)	13	0	0	

Note: Zeros indicate that no features meeting these criteria were detected.

Table S3. Genome assembly and annotation statistics for the yeast isolates INTA AB1-1 and INTA AB1-4. Assembly metrics include contiguity parameters, genome size, GC content, and repeat composition, while annotation features summarize BUSCO completeness and contamination estimates, as well as the total number of predicted genes and Gene Ontology (GO) terms.

	***Hyphopichia burtonii* strain INTA AB1-1**	***Debaryomyces fabryi* strain INTA AB1-4**
	Assembly features	
N50 (bp)	135982	286720
L50	27	14
No. of Contigs (>500 bp)	184	152
Largest contig (bp)	540439	824627
Genome Size (Mbp)	12.41	11.72
GC content	34.92	35.60
	Masked repeats	
Total TEs	0.05 %	0.00 %
SINEs	0.00 %	0.00 %
LINEs	0.04 %	0.00 %

LTR Elements	0.00 %	0.00 %
DNA Elements	0.01 %	0.00 %
Unclassified	0.00 %	0.00 %
Simply Repeats	1.99 %	0.78 %
Low Complexity	0.47 %	0.16 %
Annotation Features		
BUSCO Completeness	98.5%	98.8%
BUSCO Contamination	0.1%	0.2%
Total No. of Genes	5778	5815
Total No. of GOs	1951	1963

Table S4. Functional genomic annotation summary of the yeast isolates INTA AB1-1 and INTA AB1-4.

Feature	INTA AB1-1	INTA AB1-4	Tool / Database
Proteins with InterPro hit (%)	91.58	98.18	InterProScan
Proteins with Pfam domain (%)	86.46	92.47	
Unique InterPro entries detected (n)	6780	6745	
Unique Pfam domains detected (n)	3283	3328	
Transporter proteins detected (n)	73	96	Transporters (TCDB)
MFS (n)	36	63	
ABC (n)	0	0	
Sugar transporters (n)	30	48	
Amino acid/peptide transporters (n)	16	13	
Unique gut stress/tolerance-associated proteins (n)	456	358	Curated gut-related functional categories (InterProScan-derived; this study)
Proteins with ≥2 stress-related categories (n)	36	28	
Predominant stress category	Oxidative stress response	Oxidative stress response	
Microbial interaction-associated proteins (n)	88	87	

Adhesion / lectin / flocculin (n, %)	55 (62.5%)	58 (66.67%)	
Cell wall / surface anchor (n, %)	21 (23.9%)	15 (17.24%)	
Other interaction (n, %)	12 (13.6%)	14 (16.09%)	
Orthologous clusters (total, n)	5544	5568	Orthology (OrthoVenn3; pairwise comparison vs. closest type strain)
Shared clusters (n)	5526	5553	
Unique clusters in AB1-1 (n)	10	------------	
Unique clusters in AB1-4 (n)	------------	9	
Singleton proteins (n)	209	162	
CAZyme genes (total)	721	753	CAZymes (dbCAN)
CAZyme families detected (n)	140	142	
GH genes (% of CAZyme genes)	48.96	49.4	
Auxiliary activities genes (n)	55	57	
CBM families detected (n)	12	12	
Biosynthetic gene clusters (total)	5	5	fungiSMASH
Biosynthetic gene clusters associated with known insecticidal or entomopathogenic activity (n)	0	0	

*Note: Unique InterPro and Pfam entries correspond to non-redundant accessions identified across the predicted proteome; individual proteins may contain multiple domains.

*Microbial interaction-associated proteins: Counts correspond to unique predicted proteins; multiple domains per protein were collapsed prior to quantification.
